# Structural Transition and Antibody Binding of EBOV GP and ZIKV E Proteins from Pre-Fusion to Fusion-Initiation State

**DOI:** 10.3390/biom8020025

**Published:** 2018-05-10

**Authors:** Anna Lappala, Wataru Nishima, Jacob Miner, Paul Fenimore, Will Fischer, Peter Hraber, Ming Zhang, Benjamin McMahon, Chang-Shung Tung

**Affiliations:** 1 Los Alamos National Laboratory, Los Alamos, NM 87545, USA; vernon.anna@cantab.net (A.L.); w.nishima@lanl.gov (W.N.); jcminer@lanl.gov (J.M.); paulf@lanl.gov (P.F.); wfischer@lanl.gov (W.F.); phraber@lanl.gov (P.H.); mcmahon@lanl.gov (B.M.); 2 New Mexico Consortium, Los Alamos, NM 87545, USA; 3 College of Public Health, University of Georgia, Athens, GA 30602, USA; mzhang01@uga.edu

**Keywords:** EBOV GP, ZIKV E, pre-fusion-to-fusion transition, antibody binding

## Abstract

Membrane fusion proteins are responsible for viral entry into host cells—a crucial first step in viral infection. These proteins undergo large conformational changes from pre-fusion to fusion-initiation structures, and, despite differences in viral genomes and disease etiology, many fusion proteins are arranged as trimers. Structural information for both pre-fusion and fusion-initiation states is critical for understanding virus neutralization by the host immune system. In the case of Ebola virus glycoprotein (EBOV GP) and Zika virus envelope protein (ZIKV E), pre-fusion state structures have been identified experimentally, but only partial structures of fusion-initiation states have been described. While the fusion-initiation structure is in an energetically unfavorable state that is difficult to solve experimentally, the existing structural information combined with computational approaches enabled the modeling of fusion-initiation state structures of both proteins. These structural models provide an improved understanding of four different neutralizing antibodies in the prevention of viral host entry.

## 1. Introduction

Enveloped viruses employ a common mechanism to enter the host cell [1]. The first steps, receptor binding and membrane fusion, are initiated by the envelope protein [2,3,4]. While specific details vary among different viruses, the envelope proteins invariably go through a large conformational change [5,6] before initiating membrane fusion. These large conformational changes allow the envelope protein to assume an extended fusion-initiation conformation: the envelope protein in the fusion-initiation state is able to bridge across the viral and the host membranes, subsequently bringing the two membranes into close proximity and starting the fusion process [7,8]. Viral neutralization by antibodies may involve binding to the fusion-state structure or inhibiting its formation. Therefore, viral envelope proteins are important foci for the development of vaccines and therapeutics. Recent intense research focus on the Ebola and Zika viruses has provided new data for the structural modeling of these transitions.

Structural data for a number of viral envelope proteins are available in the Protein Data Bank (PDB) [6,9]. Many of these known structures correspond to envelope proteins in the pre-fusion state, and some of the fusion-state structures only correspond to a partial molecule (usually in a low-pH environment). To date, there are no structures for complete viral envelope proteins in the fusion-initiation state; understanding the mechanics of the conformational change from the pre-fusion to the fusion-initiation state requires such a description.

Directly determining fusion-state structures for complete viral envelope proteins by experimental methods is difficult; molecular modeling offers a readily applicable alternative means to structural characterization. We describe the use of a knowledge-based methodology (homology modeling) to develop structures of viral envelope proteins in the fusion-initiation state. We further extend the basic idea of homology modeling to include a simple concept, “proteins and protein domains that fold similarly interact similarly”, as a result, developing structural models of envelope protein–antibody complexes. In this work we focus on envelope proteins from Ebola and Zika viruses. Ebola virus causes Ebola hemorrhagic fever, a severe and highly lethal infection: the 2013–2015 West African Ebola virus epidemic (December 2013–2015) resulted in approximately 11,000 confirmed deaths and 28,000 suspected cases [10]. Zika virus is a member of the virus family Flaviviridae [11,12] that includes the Dengue virus (DENV) and West Nile virus; in contrast to the above-mentioned Ebola virus, Zika virus causes a brief, relatively mild illness, but it has been linked to congenital microcephaly and Guillan–Barré Syndrome in humans [13,14]. In mouse models, Zika virus causes microcephaly [15], as well as damage to the male reproductive system [16] and to adult neural stem cells [17]. At the time of publication, 84 countries, territories, and subnational areas reported Zika transmission [18]. The Ebola and Zika viruses represent persistent threats to public health; there are limited options available for the treatment or prevention of either virus. In this paper, we present the following models for Ebola virus glycoprotein (EBOV GP) and Zika virus envelope protein (ZIKV E):

1. A trimer model of EBOV GP in the fusion-initiation state with the Niemann–Pick C1 (NPC1) receptor and neutralizing antibodies.
2. A trimer model of ZIKV E in the fusion-initiation state with neutralizing antibodies and the surrounding 9-mer structure of ZIKV E in the pre-fusion state with neutralizing antibodies.

Our modeling approach is general and comprehensive and can be used for developing structures of other pathogen proteins in their functional states for understanding their functions; the developed structure-based knowledge can further add to sequence-based information and improve vaccine design for viruses and other pathogens. Importantly, all of the developed models are testable experimentally, potentially leading to the discovery of new targets for drugs and vaccines and the optimization of known vaccine and drug targets.

**Figure 1:**
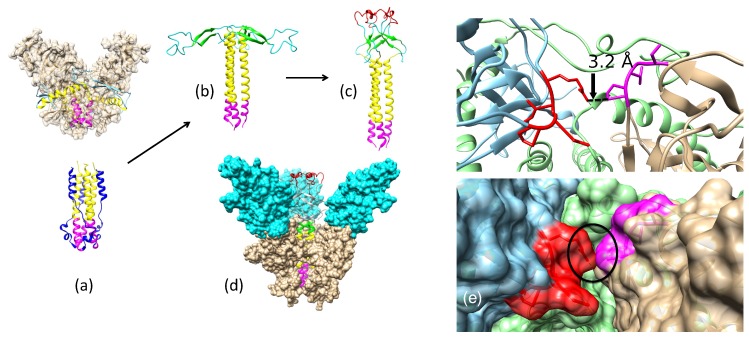
Structures of Ebola virus glycoprotein (EBOV GP) corresponding to the pre-fusion and fusion-initiation states. (**a**) (**Top**) A trimer structure of GP1/GP2 in the pre-fusion state and (**Bottom**) GP2 in the fusion-initiation state were found using X-ray crystallography (Protein Data Bank (PDB) accession codes 3CSY and 2EBO, respectively). (**b**) Model of the pre-fusion structure of the GP2 trimer with a spring-loaded mechanism. (**c**) Hairpin loop structure (residues 510–559) of GP2, including the fusion peptide (residues 524–539, *red*), remodeled into a conformation with the fusion peptide pointing toward the host cell surface. (**d**) GP1/GP2 trimer in the fusion-initiation state bound to the Niemann–Pick C1 (NPC1) receptor. Viral membrane is oriented at the bottom of each panel. (**e**) The amino group of R130 and $C_{\beta }$ of P93 are in direct contact. Loops 90–93 are shown in red, 126–130 are in magenta, GP2 is in light green, and two of the GP1 molecules are in gold and cyan. The view is from the top of the trimer. The two figures are in ribbon- and space-filling models. These contacts need to open-up to allow the long GP2 helix to go from the outside to the inside of the GP1 trimer.

Ebola virus has a small (∼18–19 kb) negative-stranded RNA genome [19] that encodes eight viral proteins. Envelope glycoprotein (GP) is the viral surface protein responsible for host cell entry [20,21], and it has a sequence of 676 residues in all known Ebola strains. The N-terminus (residues 1–32) forms a signal peptide, and the remaining protein residues (33–676) are collectively referred to as the GP portion. The host endoprotease furin cleaves GP into two segments: GP1 (residues 33–501) and GP2 (residues 502–676) [22]. GP1 is responsible for receptor binding to the host cell [23,24], while GP2 acts as a class I viral fusion protein [25]. Several functional domains of EBOV GP are denoted in Figure 1. Like influenza and HIV envelope proteins, EBOV GP is a homotrimer in its functional state [26].

Since the 2015 epidemic, significantly more Ebola virus sequence information has become available, including sequences of GP (more than 1000 unique EBOV GP sequences can be found in the current NCBI Ebola virus database) [27]. Significant efforts have been devoted towards solving the GP structure using various experimental approaches, including X-ray crystallography [26,28,29,30], NMR [31,32], and electron microscopy [33,34]. Through this work, domain structures have been solved in different functional states, and as has the trimer structure of the EBOV GP mucin-like region deletion mutant (GPmuc) in the pre-fusion state [26]. All of these pieces of information contribute to a basis for developing a structural model of the GP in the fusion-initiation state. Zika virus possesses a non-segmented, single-stranded, positive-sense RNA genome (10 kb) [12]. The urgency in finding ways to combat the virus has increased significantly since a causal relation between Zika virus and the apoptosis of human neurons was discovered [13]. For EBOV, the envelope protein (GP) forms homotrimers that are distributed sparsely on the surface of the virus, as in influenza and HIV [26]. By contrast, ZIKV E’s form homodimers and cover the entire viral surface (the capsid comprises 90 homodimers, arranged as 30 rhombic faces with 3 dimers each) [12,35]. However, the partial structure of ZIKV E in the fusion-initiation state (at low pH) shows a trimer arrangement [36]. Determining how ZIKV E transforms from a pre-fusion dimer to a fusion-state trimer presents a considerable challenge to the structural modeling community. Although ZIKV E and EBOV GP are phylogenetically distinct viruses, their fusion subunits both adopt a trimer structure; in this study, we investigate some of the similarities, differences, and consequences of these fusion-state structures.

## 2. Results

### 2.1. Pre-Fusion and Fusion-Initiation State Structures of EBOV GP (“Spring-Loaded Model”)

The crystal structure of EBOV GP1/GP2 has been described in the pre-fusion state (accession code 3CSY) [26]. In this structure, GP1 and GP2 exist as a trimer, with the GP2 bound from the outside and the inside of the GP1 proteins. A partial structure of GP2 (lacking N- and C-terminal domains) in the fusion-initiation state has also been solved at low pH (accession code 2EBO) [28]. The pre-fusion and fusion-initiation states of GP2 show different folds (see Figure 1a) with a small triple-helix structure shown in both (highlighted in magenta).

In the pre-fusion structure, the yellow/magenta regions of GP2 are wrapped around the GP1 trimer on the outside of the GP1 trimer. For the yellow helices to stack on top of the magenta helices as indicated (Protein Data Bank (PDB) accession code 2EBO), they must rearrange from the outside to the inside of the GP1 trimer. A close inspection of the GP structure in the pre-fusion state reveals a connection between two GP1 proteins in the trimer through loops S90 to P93 and P126 to R130. To accomplish the transition step, this weak bridge needs to be disconnected to allow the N-terminal part of GP2 to move from the outside of the GP1 trimer to the inside.

The C-terminal region of GP2 (highlighted in blue, Figure 1a) needs to “peel off” from the central triple helix of the fusion-initiation state in order for GP2 to bind and fit into the internal part of the GP1 trimer. Because the C-terminal end of GP2 contains the trans-membrane domain that points toward the viral surface—and is less likely to be immunogenic—our model is truncated at residue 599. To account for the large conformational changes during the pre-fusion-to-fusion transition, a spring-loaded model is applied [37,38]. This same model was considered as the mechanism for the EBOV GP structural transition in a previous study by White et al. [39] and is supported by structural information of EBOV GP in two different states (Figure 1a). The spring-loaded model suggests that the two GP2 helices in the pre-fusion state structure (3CSY) should be stacked together to form one long helix, as shown in the fusion-initiation-state structure (2EBO). When the two helices become one long helix, the N-terminal region of GP2 (residues 502–555) will be stacked on top of the long triple helix and pointed radially outward (Figure 1b).

The known structures of viral envelope proteins in the fusion-initiation state usually show a trimer arrangement in a compact state [6], where the fusion peptides are tightly localized on top of the trimer and are exposed to the host cell membrane [36]. The GP2 structure (Figure 1c) has the fusion peptides (residues 514–539, highlighted in red) separated and aligned outwards, parallel to the viral membrane, instead of upward toward the host cell membrane. In order to develop a model that matches known fusion-initiation-state structures, the sole β-hairpin structure in GP2 (residues 517–520 and 543–546) are fitted to a trefoil fold β-hairpin template (PDB accession code 2F2F) at each trimer (Figure A2). The modeled β-hairpins in the trefoil fold are then docked on top of the yellow triple helix using the disulfide bond between C511 and C556 as a constraint. Finally, the low-pH fusion loop structure (PDB accession code 2RLJ) is used as a template to model the fusion peptide structure (residues 521–542). The final structure of the GP2 trimer in the fusion-initiation state is shown in Figure 1c.

NPC1 has been identified as the entry receptor for EBOV GP [40], which must be primed to a fusion-competent state before binding to a NPC1 receptor. The priming process includes the cleavage of the mucin-like domain, the glycan cap, and the outmost strand of the proposed receptor binding region [26,41]. Using the NPC1-bound GP1 structure (PDB accession code 5F1B) as a template, the primed GP1–GP2–NPC1 complex in the fusion-initiation state was modeled (Figure 1d).

On the basis of these results, we propose that the connecting bridge in the trimer (S90–P93 and P126–R130) serves as a gate for the pre-fusion-to-fusion structural transition. If this gate is locked, the structural transition step is inhibited, which can possibly impede viral replication.

### 2.2. Pre-Fusion and Fusion Structure of ZIKV E

**Figure 2:**
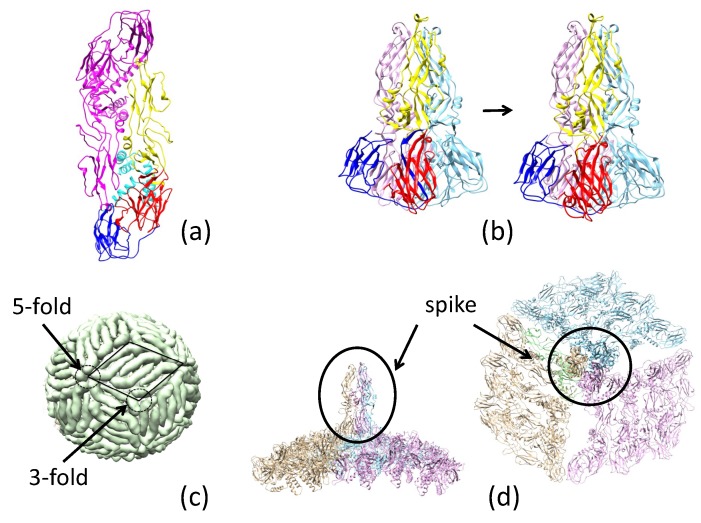
The pre-fusion and fusion structures of the Zika virus envelope protein (ZIKV E). (**a**) The 3.8 Ångström resolution cryo-EM structure of the ZIKV E dimer (Protein Data Bank (PDB) accession code 5IRE). (**b**) *Left*: Partial structure of Dengue virus (DENV E) (PDB accession code 1OK8). *Right*: Modeled structure of ZIKV E in its fusion-initiation states. These structures are divided into four domains, highlighted as follows: I: red, II: yellow, III-1: blue, and III-2: cyan. (**c**) The 180 copies of ZIKV E (from cryo EM, PDB accession code 5IRE) cover the surface of the viral capsid, with intersection points of 3- and 5-fold symmetries marked. (**d**) A model of side and top views of the ZIKV E trimer (marked as a spike) in the fusion-initiation state.

The basic unit of ZIKV E, on the basis of structures derived from X-ray crystallography and cryo-EM (PDB accession codes 5JHM and 5LBS), shows the protein in a head-to-tail dimer arrangement (Figure 2a). While the structure of ZIKV E in the fusion-initiation state is not known, the partial ZIKV E structure of the related Dengue virus (DENV) has been described in its fusion-initiation state (PDB accession code 1OK8). As a result of the high sequence similarity of DENV and ZIKV E, it is a simple exercise to model the structure of ZIKV E in the fusion-initiation state using the homology modeling approach (Figure 2b).

On the basis of the known structure, ZIKV E can be divided into three domains (Figure A3): domain I (residues 1–52, 132–193, and 280–296), domain II (residues 53–131 and 193–276), and domain III (residues 297–501). In this work, we further divide domain III into domain III-1 (residues 297–403) and domain III-2 (residues 404–501). Domain III-2 is absent in the fusion-initiation-state structure. Converting ZIKV E from 2- to 3-fold symmetry is a significant modeling challenge. Schematic representations of the flavivirus membrane-fusion mechanism have been proposed [42,43], involving (i) a low-pH induced dissociation of the ZIKV E dimer, (ii) outward projection of the ZIKV E monomer, and finally, (iii) the formation of the fusion-state trimer. The proposed process does not provide information about the full ZIKV E structure in the fusion-initiation state, and thus the relative position and orientation of the trimer with respect to the rest of the ZIKV E monomers on the surface of the virus require further elucidation. Here, we propose a direct path for the ZIKV E pre-fusion-to-fusion structural conversion.

The cryo-EM structure of ZIKV E (PDB accession code 5IRE) is shown in Figure 2c. In this representation, 3 dimers form a rhombus-shaped hexamer (highlighted by the parallelogram), and 30 copies of this hexamer are sufficient to cover the entire surface of the virus. These hexamers form 3- and 5-fold symmetries at their vertices, where the ends of either three or five ZIKV E’s meet. The basic unit used to model the pre-fusion-to-fusion transition consists of three dimers that form a 3-fold symmetry. Because domain III-2 is absent in the DENV E protein fusion-initiation-state structure, we propose that this region serves as an anchor for the structural transition and that domains I, II, and III-1 from each of the three ZIKV E molecules are involved in the structural transition. This transition is accomplished through a 90${}^{\circ }$ rotation of domain I/II. The binding modes of I/II and III-1 are different between the pre-fusion (3CSY) and fusion (1OK8-derived) structures. The I/II and III-1 binding mode for 1OK8 upsets the connection between III-1 and III-2; thus the I/II/III-1 arrangement of the pre-fusion structure is used in the final fusion-initiation state (spike in Figure 2d).

### 2.3. Neutralizing Antibody Blocks Viral Entry by Pre-Fusion and Fusion-Initiation-State Interactions

Neutralizing antibodies inhibit pathogen entry into the host cell. Because of the fact that this is a complex process [44], the blocking of viral host-cell entry may occur at multiple stages. Because the conformational transition of the viral envelope protein from a pre-fusion to a fusion-initiation state is a crucial step in host-cell entry, we explored different neutralizing antibodies and their roles in blocking cell entry by studying the binding of antibodies to ZIKV E in pre-fusion and fusion-initiation states.

#### 2.3.1. Antibody KZ52 Blocks EBOV Entry by Preventing GP Structural Transition from a Pre-Fusion to a Fusion-Initiation State

The KZ52-bound Zaire-EBOV GP trimer in the pre-fusion state has been found using X-ray crystallography (PDB accession code 3CSY). Residues of the GP that are responsible for KZ52 binding include GP1 (residues 42/43) and GP2 (residues 505–511/513–514/549–553/556). If the N-terminal portion of GP2 cannot peel off from the outside and move to the inside of the GP1 trimer, then the conformational transition to the fusion-initiation-state structure cannot be completed. Therefore, the binding of KZ52 to EBOV GP possibly prevents the structural transition of the protein to the fusion-initiation state. A similar binding mechanism was observed for the binding of 16F6 to Sudan EBOV GP (PDB accession code 3S88). Therefore, we may argue that 16F6 blocks cell entry by preventing the structural transition of GP from a pre-fusion to a fusion-initiation state.

#### 2.3.2. Antibody mAb100 Blocks EBOV Entry through Two Different Mechanisms

**Figure 3:**
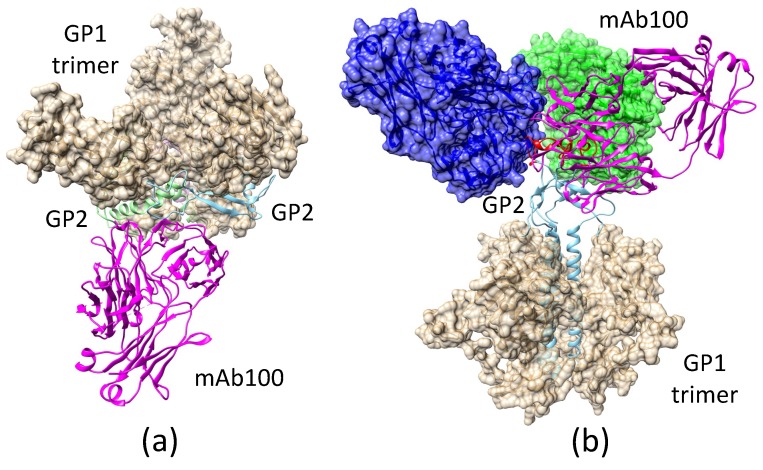
The mAb100 antibody can bind to the Ebola virus glycoprotein (EBOV GP) trimer in both pre-fusion and fusion-initiation states. (**a**) Modeled mAb100 binding to two GP2 monomers when the GP trimer is in the pre-fusion state, preventing the fusion-initiation state transition. (**b**) A post-transition model, in which the mAb100 binding site on GP2 is exposed at the top of the trimer, allowing the antibody to bind. The model shows that up to three mAb100 antibodies can bind to the GP trimer in the fusion-initiation-state structure without steric interference.

The antibody mAb100, when used in conjunction with mAb114, can protect non-human primates against all signs of Ebola virus disease [45]. The crystal structure of mAb100-bound EBOV GP (PDB accession code 5FHC) shows that mAb100 binding occludes access to the cathepsin-cleavage loop (Figure 3), thus preventing viral entry. It is a straightforward exercise to develop a model of an EBOV GP trimer with bound mAb100 by superimposing the GP structure in 5FHC with that in 3CSY. In this model, mAb100 interacts with two copies of GP2 in the trimer. As a result, mAb100 directly blocks the transition from the pre-fusion to the fusion-initiation state. From the perspective of 5FHC, mAb100 recognizes and binds to a loop near the fusion peptide in GP2. Using this binding mode, we developed a model of mAb100 binding to a GP trimer in the fusion-initiation state. It was further shown that up to three mAb100 antibodies can bind simultaneously to a GP trimer in the fusion-initiation state. While the fusion peptide is exposed when the GP trimer is in the fusion-initiation-state structure, the exposure is blocked when mAb100 binds to the viral GP.

#### 2.3.3. Antibody 2A0G6 Binds to ZIKV E in the Fusion-Initiation State and Blocks the Exposure of the Fusion Peptide

**Figure 4:**
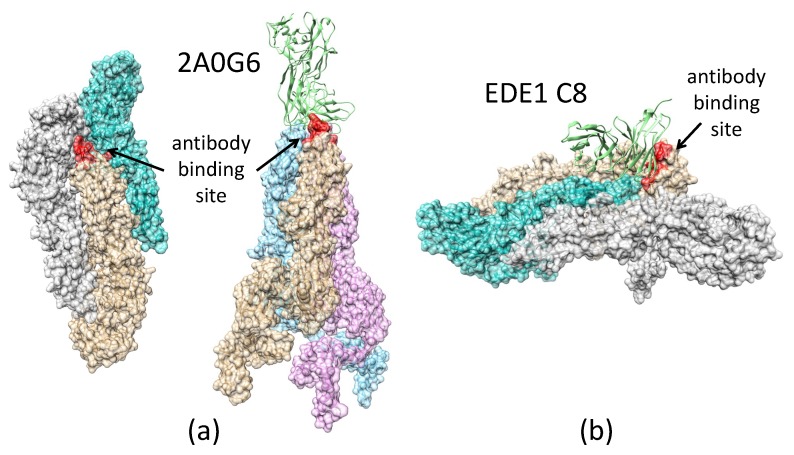
Two different antibodies, 2A0G6 (PDB accession code 5JHL) and EDE1 C8 (PDB accession code 5LBS), interact with the Zika virus envelope protein (ZIKV E) to prevent viral cell entry with different mechanisms. In this figure, the antibody recognition site is colored in red. (**a**) ZIKV E in the pre-fusion state: the antibody binding site is partially buried and prevents binding of 2A0G6 to ZIKV E. (**b**) ZIKV E in the fusion-initiation state: antibody binding site is exposed, allowing 2A0G6 to bind to the E protein. The antibody EDE1 C8 binds to two copies of the ZIKV E and prevents transition from the pre-fusion to fusion-initiation state.

The structure of ZIKV E, bound with a broadly protective antibody 2A0G6 (Figure 4a), was found using X-ray crystallography (PDB accession code 5JHL) [46]. The structure shows that the antibody interacts with ZIKV E residues 76, 77, and 105–108. These residues lie on the tip of the GP close to the fusion loop. In the pre-fusion state, these residues are buried and cannot be reached by the antibody, but in the fusion-initiation state, these residues become exposed. When the antibody binds, the fusion loop (residues 100–108) is blocked from exposure, and the fusion process is blocked.

#### 2.3.4. Antibody EDE1-C8 Blocks the Pre-Fusion to Fusion Transition of ZIKV E and Prevents Cell Entry

The antibody EDE1-C8 binds to the pre-fusion-state structure of ZIKV E on the surface of the virus, as shown in Figure 4 and Figure A4. With the antibody bound, it prevents the peeling off of the N-terminal regions (I, II, and III-1) of the ZIKV E protein from its neighboring ZIKV E as well as the formation of the fusion-state trimer structure. With the ZIKV E locked in the pre-fusion structure, it prevents the pre-fusion-to-fusion transition.

#### 2.3.5. Videos Describing the Transitions from the Pre-Fusion to Fusion Structures

Large conformational changes are associated with the pre-fusion-to-fusion-initiation-state structures of the viral envelope protein. Although the structures at both ends of the transition are known, it is not clear how the structures transform from one to the other. To illustrate the complex process, two videos as an artist’s representation of the transition were developed (Video S1, Video S2). The two videos depict a possible (but not exclusive) transition pathway for EBOV GP and ZIKV E. In these videos, the integrity of the stereochemistry was maintained for the structures in each frame. Many of the features associated with the transition and that are described in the manuscript are easily identifiable in the videos.

## 3. Discussion

Viral cell entry is a complex process involving multiple molecular participants and conformational changes. We provide a plausible mechanism in atomic detail for the pre-fusion-to-fusion-state transition for both the Ebola and Zika viruses. While these two viruses are very different, their fusion proteins share common features in the initiation of cell entry. The most apparent common feature is a trimer arrangement of the proteins in the fusion-initiation state. This can be seen in other viruses, such as influenza and HIV. Secondly, these fusion proteins undergo large conformational changes to transition from the pre-fusion- to fusion-initiation-state structures. To prevent viral cell entry, antibodies can act on the fusion protein using two different mechanisms: (1) preventing the structural transition, and (2) blocking fusion peptide access to the cell membrane.

**Figure A1:**
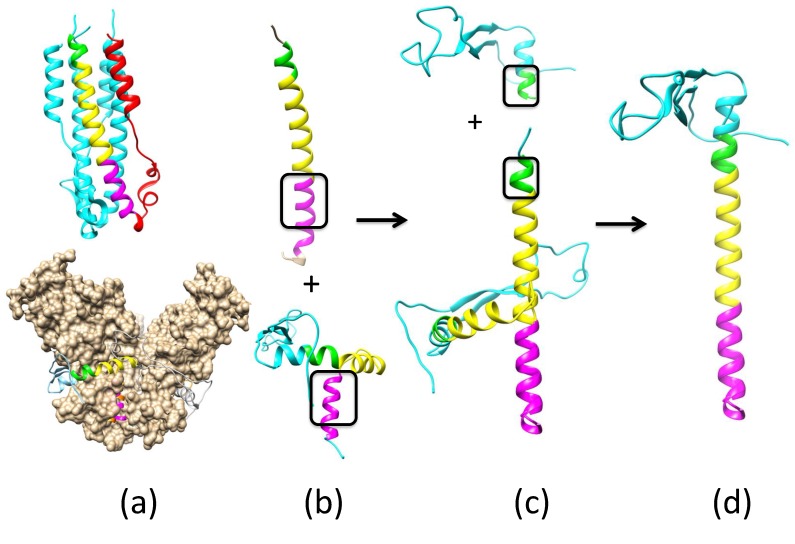
(**a**) Incomplete crystal structures of Ebola virus glycoprotein (EBOV GP) in its pre-fusion state (**above**) and fusion state (**below**). (**b**) Modeled triple helix formed by segments (residues 583–595) boxed and highlighted in magenta. (**c**) N-terminal residues (514–564, color-coded in cyan and green) of GP2 from 3CSY added to the long helix by matching the green segments of the helices. (**d**) The resulting complete model of GP2 in the fusion state.

**Figure A2:**
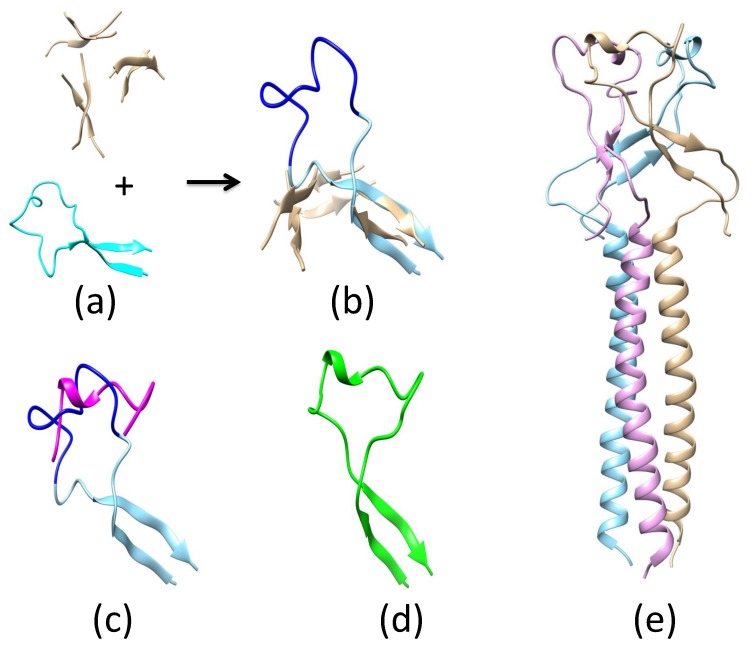
(**a**) Residues 514–559 (**below**). Protein Data Bank analysis shows that the structure of a β-hairpin trefoil fold can be found in the crystal structure of the cytolethal distending toxin (CDT) (**above**). (**b**) Remodeled residues 514–559 of GP2 in the fusion-state structure adopt a β-hairpin structure. These three β-hairpins form a trefoil fold. (**c**) The NMR structure of the fusion loop (residues 525–538) is shown in red. The known structure of the fusion loop was substituted into the corresponding region in the hairpin (shown in blue). (**d**) The remodeled structure of the hairpin. (**e**) The resulting complete model of the GP2 in the fusion state.

**Figure A3:**
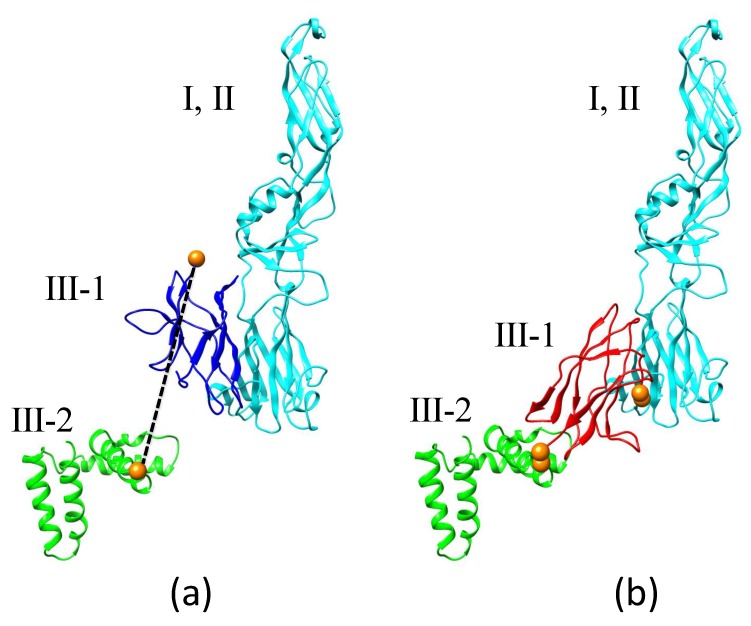
(**a**) Zika virus envelope protein (ZIKV E) domains I and II (residues 1–304) are shown in cyan, domain III-1 (residues 305–403) is shown in blue, and domain III-2 (residues 404–501) is shown in green. (**b**) ZIKV E domains I and II (residues 1–304) are shown in cyan, domain III-1 (residues 305–403) is shown in red, and domain III-2 (residues 404–501) is shown in green.

**Figure A4:**
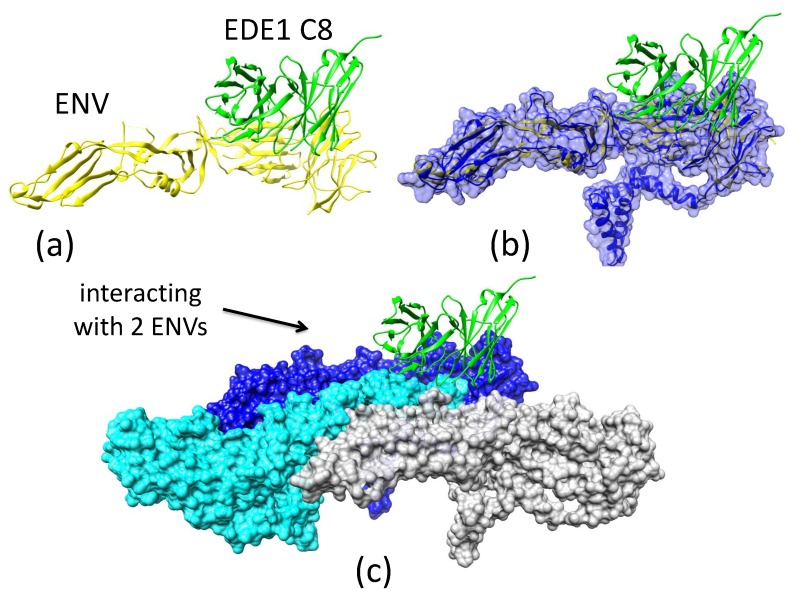
(**a**) The crystal structure of EDE1 C8/ZIKV E complex (Protein Data Bank (PDB) accession code 5LBS) is shown as a ribbon model. (**b**) After matching ZIKV E in the virus/antibody complex to one of the Zika monomers on the surface of the virus, the antibody could be positioned as shown. (**c**) EDE1 C8 interacting with two ZIKV E’s (colored in blue and cyan).

With the known structures of these proteins in the pre-fusion state and partial structures of the proteins in the fusion-initiation state (Figure A1), we developed near-complete fusion-state structural models of EBOV GP and ZIKV E. Using a spring-loaded mechanism proposed for influenza hemagglutinin (HA) structural transition, the fusion-initiation-state trimer structure of EBOV GP was developed. The fusion-initiation-state trimer structure of ZIKV E was developed from three dimers on the surface of the virus by utilizing the 3-fold symmetry.

Combining the information of EBOV GP/ZIKV E structures in both the pre-fusion and the fusion-initiation states, as well as the structures of these proteins in complexes with different neutralizing antibodies, we investigated the mechanisms used by the antibodies to prevent the process of viral cell entry. We found that KZ52 (Ebola) and EDE1-8 (Zika) prevented cell entry by blocking the pre-fusion-to-fusion structural transition. We propose that the connecting bridge in the GP1 trimer (S90–P93 and P126–R130) serves as a gate for the pre-fusion-to-fusion structural transition: if this gate is locked, the structural transition step is inhibited, and the viral life cycle is halted. We also demonstrated that the antibody 2A0G6 binds to ZIKV E in the fusion-initiation state and possibly prevents the fusion peptide from reaching the cell membrane. The antibody mAb100 is able to bind EBOV GP in both the pre-fusion and the fusion-initiation states, preventing viral cell entry by both inhibiting the structural transition and blocking fusion peptide access to the cell membrane.

With the collective efforts to expand the information base in both biological sequences and structures, GenBank and PDB provide invaluable information. This information enables the expansion of structural modeling to a new realm that includes proteins in higher-order structures and/or transitions. Using EBOV GP and ZIKV E as examples, we have demonstrated that a conceptually straightforward, knowledge-based approach allows us to develop models of the fusion-initiation-state structures for both proteins in their functional units. With this information, we were able to study the binding of neutralizing antibodies to these proteins and propose the exact mechanisms for how these antibodies block cell entry to stop viral replication.

## 4. Materials and Methods

### 4.1. Homology Modeling Using a Motif-Matching Fragment Assembly Method

The MMFA was used for complex homology modeling (described in our earlier work [47]). This approach was used to develop structural models of neutralizing antibodies binding to their targeted fusion proteins in their functional unit and state.

### 4.2. Structure Alignment and Superposition

To develop the fusion-initiation-state structure of the GP protein using the spring-loaded mechanism, domain structural assembly was accomplished by superposition of structurally matched regions (e.g., helical segments). The MatchMaking option in the Structural Comparison Tool of Chimera [48] was used for the structure alignment and superposition of the structurally matched segments.

### 4.3. Structural Refinement

Molecular dynamics simulations of Zika and Ebola models were performed in a standard manner as described in Hess et al. [49]. Initially, structures were energy-minimized with a steepest-descent minimization algorithm, after which the protein underwent a 100 ps NVT (constant number of particles N, volume V, and temperature T) equilibration step, followed by NPT (constant number of particles N, pressure P, and temperature T) equilibration for another 100 ps. The production run was performed under standard NPT conditions at 300 K for 100 ns.

### 4.4. Graphics

Molecular graphics images were produced using the UCSF Chimera package [50] from the Resource for Bio-computing, Visualization and Informatics at the University of California, San Francisco.

## 5. Conclusions

In conclusion, we investigated the structure–function relationship of EBOV GP and ZIKV E’s. While the two viruses are phylogenetically distinct, their envelope proteins share common structural features in the initiation of cell entry. Combining the available structural information from both low- and high-resolution structures, we used a computational approach developed in our laboratory that included the utilization of the Motif-Matching Fragment Assembly method (MMFA) [47], allowing us to generate atomic-level models of EBOV GPs and ZIKV E’s in the fusion state. These models, together with the knowledge of the proteins in the pre-fusion state, allowed us to study the function of four neutralizing antibodies as they bind to the viral envelope proteins. We found that the antibodies KZ522 (Ebola virus) and EDE1-C8 (Zika virus) prevent cell entry by blocking the pre-fusion-to-fusion transition. The antibody 2A0G6, on the other hand, prevents the exposure of the fusion peptide when ZIKV E is in the fusion state. The antibody mAb100 can make use of both mechanisms by blocking the transition and the exposure of the fusion peptide when Ebola GP is in the fusion state. We demonstrated a possible pathway from pre-fusion to fusion states for both EBOV GPs and ZIKV E’s in two very different structural arrangements. The modeling approach described in this work provides a framework for developing structure-based knowledge that can improve the understanding of sequence–structure relationships and of protein–protein interactions and functions, which can aid vaccine design for viruses and other pathogens.

## Supplementary Materials

The following are available online at http://www.mdpi.com/2218-273X/8/2/25/s1: Video S1: EBOV GP transition model; Video S2: ZIKV E transition model.

## Appendix A

### Appendix A.1. Modeling Fusion-State Structure of EBOV GP Using the Spring-Loaded Mechanism

The crystal structures of EBOV GP in the fusion and pre-fusion states are shown in the top and bottom images in Figure A1, respectively. Neither of the structures is complete; however, the pre-fusion GP structure (3CSY) is significantly more complete and includes large portions of GP1 (residues 31–310) and GP2 (residues 502–595). Only a partial structure of GP2 (residues 557–630) is solved and is available to the public (2EBO). The color coding of the helical regions corresponds to the spring-loaded mechanism with the following color assignments: green: 560–564; yellow: 565–582; magenta: 583–595.

In the fusion-state structure of the GP trimer, the C-terminal regions (residues 596–603, highlighted in red) are wrapped around the triple helices formed from the N-terminal regions (residues 557–595) of the three GP2 molecules. In the pre-fusion structure of the GP trimer, only a small portion of GP2, the triple helix formed from the magenta-colored helices, is internal to the GP1 trimer (shown in a space-filling model in Figure A1a). The remaining parts of GP2 are wrapped around the GP1 trimer from the outside. With the central cavity in the GP1 trimer (3CSY) being limited and only being able to accommodate a tight triple helix, the C-terminal regions of GP2 (red helix in Figure A1a) have to be “peeled” from the central triple helix and moved to the space below the triplex. With this part of the molecule including the membrane proximal external region (MPER) and the transmembrane helix (TM), its main function should be to anchor the protein to the surface of the virus. With insufficient information to model the structure of this portion of the molecule, we decided to leave this segment out of our model.

The common structure between the pre-fusion (3CSY) and fusion (2EBO) structures of GP2 is the triple helix formed by segments (residues 583–595) boxed and color highlighted in magenta in Figure A1b. By matching the magenta-colored helices, the structure of the three helical segments (magenta, yellow, and green) in 3CYS could be replaced by a single long helix from 2EBO. The N-terminal residues (514–564, color-coded in cyan and green) of GP2 from 3CSY were added to the long helix by matching the green segments of the helices (see Figure A1c), resulting in the model of GP2 in the fusion state (see Figure A1d).

### Appendix A.2. Remodeling the Fusion Loop Structure

With the modeled structure as shown in Figure A1d, in a trimer arrangement, the fusion loops point sideways instead of upwards to the host membrane. We decide to remodel this portion (residues 514–559) of GP2 in the fusion-state structure. With the segment adopting a β-hairpin structure (see Figure A2a), the task became arranging three β-hairpins into a trefoil fold. By analyzing the protein database, we learned that the structure of a β-hairpin trefoil fold can be found in the crystal structure of the cytolethal distending toxin (CDT) (PDB accession code 2F2F; see the top image of Figure A2a). By matching the two β-strands in the GP2 hairpin to those in the trefoil fold, the hairpin in a trefoil fold arrangement could be modeled, as shown in Figure A2b. The NMR structure of the fusion loop (residues 525–538) has been solved and is available in PDB (accession code 2RLJ; shown as the red molecule in Figure A2c). We substituted this known structure of the fusion loop into the corresponding region in the hairpin (colored blue in Figure A2c) and obtained the remodeled structure of the hairpin as shown in Figure A2d. The final model of the GP2 trimer in the fusion state is shown in Figure A2e.

### Appendix A.3. Determination of ZIKV E Domain III-1 Placement in the Fusion-State Structure

It is clear that domains I and II (residues 1–304, colored in cyan in Figure A3a,b) need to rotate 90${}^{\circ }$ to transit from the pre-fusion to the fusion state. It is also clear that domain III-2 (residues 404–501, colored in green in Figure A3a,b) should serve as the anchor for ZIKV E in the fusion state and should be connected to other E proteins on the surface of the virus. Placing the domain III-1 (residues 305–403) relative to other domains in ZIKV E when the protein is in the fusion state is a problem that needed to be addressed: in the first case, when domains I and II move into the fusion-state structure, domain III-1 can move together with domains I and II (as shown in Figure A3a); in the second case, domain III-1 may stay with domain III-2 and not move together with domains I and II (as shown in Figure A3b). In these figures, the connecting residues between domains II/III-1 and III-1/III-2 are highlighted as orange spheres. One can see that in the first case (Figure A3a), the distance between the two orange spheres is ∼ 50 Å, which is too great to form a connection. If domain III-1 did not move together with domains I and II, as in the second case, the two orange spheres that connect domains II/III-1 and III-1/III-2 are separated with appropriate distances to form the connection. On the basis of this exercise, we determined that domain III-1 should stay with domain III-2 while domains I and II are rotated into a new position when ZIKV E undergoes the pre-fusion-to-fusion-state transition.

### Appendix A.4. Binding of EDE1 C8 to ZIKV E’s on the Surface of the Virus

While the crystal and solution environments are quite different, the known structures of various proteins in the two different environments have shown similar structural folds [51]. Here, we assumed that the binding mode derived from crystal structures of the protein/antibody complexes provides a stable interaction for the complex under physiological conditions. The crystal structure of the EDE1 C8/ZIKV E complex has been solved (PDB accession code 5LBS) and is shown (ribbon model) in Figure A4a. We could use this binding mode to see how the antibody interacts with ZIKV E when it is in its functional state (i.e., when the E proteins cover the entire viral surface). In this figure, three ZIKV E’s in the functional state (PDB accession code 5IRE) are shown in a space-filling model (blue, cyan, and gray). The task was to bring the ZIKV E/antibody complex to the surface of the virus. When we matched ZIKV E in the ZIKV E/antibody complex (shown in Figure A4a) to one of the ZIKV E monomers (colored in blue) on the surface of the virus, we could bring the antibody to the surface of the virus, as shown in Figure A4b. As a result of this exercise, we see that EDE1 C8 interacts with two E proteins (colored in blue and cyan), as shown in Figure A4c.

### Appendix A.5. Animation

**Video 1.** A transition path of EBOV GP between pre-fusion and fusion states (*left*: side; *right*: top). The intermediate structures were made by morphing and minimization. Each domain is colored as follows: GP1: yellow, red, and blue; GP2: orange, cyan, and green).

**Video 2.** A transition path of ZIKV E between pre-fusion and fusion states (*left*: side view; *right*: top view). To clarify the structure, Zika domains were colored as follows: I: yellow; II– red; III-1: blue; III-2: cyan; the remaining two units of the trimer are orange and green; the remaining structures are gray.

## References

[1] Eckert D.M., Kim P.S. (2001). Mechanisms of viral membrane fusion and its inhibition. Annu. Rev. Biochem..

[2] Kielian M., Jungerwirth S. (1990). Mechanisms of enveloped virus entry into cells. Mol. Biol. Med..

[3] Dimitrov D.S. (2004). Virus entry: Molecular mechanisms and biomedical applications. Nat. Rev. Microbiol..

[4] Más V., Melero J.A. (2013). Entry of Enveloped Viruses into Host Cells: Membrane Fusion.

[5] Sullivan N., Sun Y., Sattentau Q., Thali M., Wu D., Denisova G., Gershoni J., Robinson J., Moore J., Sodroski J. (1998). CD4-induced conformational changes in the human immunodeficiency virus Type 1 gp120 glycoprotein: Consequences for virus entry and neutralization. J. Virol..

[6] Riedel C., Vasishtan D., Siebert C.A., Whittle C., Lehmann M.J., Mothes W., Grünewald K. (2017). Native structure of a retroviral envelope protein and its conformational change upon interaction with the target cell. J. Struct. Biol..

[7] Tran E.E.H., Borgnia M.J., Kuybeda O., Schauder D.M., Bartesaghi A., Frank G.A., Sapiro G., Milne J.L.S., Subramaniam S. (2012). Structural Mechanism of Trimeric HIV-1 Envelope Glycoprotein Activation. PLOS Pathog..

[8] Wilen C.B., Tilton J., Doms R. (2012). HIV: Cell binding and entry. Cold Spring Harb. Perspect. Med..

[9] Berman H., Westbrook J., Feng Z., Gilliland G., Bhat T., Weissig H., Bourne P. (2000). The Protein Data Bank. Nucleic Acids Res..

[10] Ebola Data and Statistics World Health Organization Situation Summary Data Published on 31 July 2015. http://apps.who.int/gho/data/view.ebola-sitrep.ebola-summary-20150731?lang=en.

[11] Malone R.W., Homan J., Callahan M.V., Glasspool-Malone J., Damodaran L., Schneider A.D.B., Zimler R., Talton J., Cobb R.R., Ruzic I. (2016). Zika virus: Medical countermeasure development challenges. PLOS Negl. Trop. Dis..

[12] Cunha M.S., Esposito D.L.A., Rocco I.M., Maeda A.Y., Vasami F.G.S., Nogueira J.S., de Souza R.P., Suzuki A., Addas-Carvalho M., Barjas-Castro M.d.L. (2016). First complete genome sequence of Zika Virus (Flaviviridae, Flavivirus) from an autochthonous transmission in Brazil. Genome Announc..

[13] Dang J., Tiwari S.K., Lichinchi G., Qin Y., Patil V.S., Eroshkin A.M., Rana T.M. (2016). Zika virus depletes neural progenitors in human cerebral organoids through activation of the innate immune receptor TLR3. Cell Stem Cell.

[14] Rasmussen S.A., Jamieson D.J., Honein M.A., Petersen L.R. (2016). Zika virus and birth defects—Reviewing the evidence for causality. N. Engl. J. Med..

[15] Huang W.C., Abraham R., Shim B.S., Choe H., Page D.T. (2016). Zika virus infection during the period of maximal brain growth causes microcephaly and corticospinal neuron apoptosis in wild type mice. Sci. Rep..

[16] Govero J., Esakky P., Scheaffer S.M., Fernandez E., Drury A., Platt D.J., Gorman M.J., Richner J.M., Caine E.A., Salazar V. (2016). Zika virus infection damages the testes in mice. Nature.

[17] Souza B.S.F., Sampaio G.L.A., Pereira C.S., Campos G.S., Sardi S.I., Freitas L.A.R., Figueira C.P., Paredes B.D., Nonaka C.K.V., Azevedo C.M. (2016). Zika virus infection induces mitosis abnormalities and apoptotic cell death of human neural progenitor cells. Sci. Rep..

[18] Zika Data and Statistics World Health Organization Situation Summary Data Published on 10 March 2017. http://www.who.int/emergencies/zika-virus/situation-report/10-march-2017/en/.

[19] Quick J., Loman N.J., Duraffour S., Simpson J.T., Severi E., Cowley L., Bore J.A., Koundouno R., Dudas G., Mikhail A. (2016). Real-time, portable genome sequencing for Ebola surveillance. Nature.

[20] Nanbo A., Imai M., Watanabe S., Noda T., Takahashi K., Neumann G., Halfmann P., Kawaoka Y. (2010). Ebolavirus is internalized into host cells via macropinocytosis in a viral glycoprotein-dependent manner. PLOS Pathog..

[21] Aleksandrowicz P., Marzi A., Biedenkopf N., Beimforde N., Becker S., Hoenen T., Feldmann H., Schnittler H.J. (2011). Ebola virus enters host cells by macropinocytosis and clathrin-mediated endocytosis. J. Infect. Dis..

[22] Volchkov V.E., Feldmann H., Volchkova V.A., Klenk H.D. (1998). Processing of the Ebola virus glycoprotein by the proprotein convertase furin. Proc. Natl. Acad. Sci. USA.

[23] Wang J., Manicassamy B., Caffrey M., Rong L. (2011). Characterization of the receptor-binding domain of Ebola glycoprotein in viral entry. Virol. Sin..

[24] Manicassamy B., Wang J., Jiang H., Rong L. (2005). Comprehensive analysis of Ebola Virus GP1 in viral entry. J. Virol..

[25] Weissenhorn W., Carfí A., Lee K.H., Skehel J.J., Wiley D.C. (1998). Crystal structure of the Ebola Virus Membrane Fusion Subunit, GP2, from the Envelope Glycoprotein ectodomain. Mol. Cell.

[26] Lee J.E., Fusco M.L., Hessell A.J., Oswald W.B., Burton D.R., Saphire E.O. (2008). Structure of the Ebola virus glycoprotein bound to an antibody from a human survivor. Nature.

[27] Brister J.R., Bao Y., Zhdanov S.A., Ostapchuck Y., Chetvernin V., Kiryutin B., Zaslavsky L., Kimelman M., Tatusova T.A. (2014). Virus variation resource—Recent updates and future directions. Nucleic Acids Res..

[28] Malashkevich V.N., Schneider B.J., McNally M.L., Milhollen M.A., Pang J.X., Kim P.S. (1999). Core structure of the envelope glycoprotein GP2 from Ebola virus at 1.9-Å resolution. Proc. Natl. Acad. Sci. USA.

[29] Dias J.M., Kuehne A.I., Abelson D.M., Bale S., Wong A.C., Halfmann P., Muhammad M.A., Fusco M.L., Zak S.E., Kang E. (2011). A shared structural solution for neutralizing Ebolaviruses. Nat. Struct. Mol. Biol..

[30] Bale S., Dias J.M., Fusco M.L., Hashiguchi T., Wong A.C., Liu T., Keuhne A.I., Li S., Woods V.L., Chandran K. (2012). Structural basis for differential neutralization of Ebolaviruses. Viruses.

[31] Freitas M.S., Gaspar L.P., Lorenzoni M., Almeida F.C.L., Tinoco L.W., Almeida M.S., Maia L.F., Degrève L., Valente A.P., Silva J.L. (2007). Structure of the Ebola Fusion Peptide in a membrane-mimetic environment and the interaction with lipid rafts. J. Biol. Chem..

[32] Gregory S.M., Harada E., Liang B., Delos S.E., White J.M., Tamm L.K. (2011). Structure and function of the complete internal fusion loop from Ebolavirus glycoprotein 2. Proc. Natl. Acad. Sci. USA.

[33] Murin C.D., Fusco M.L., Bornholdt Z.A., Qiu X., Olinger G.G., Zeitlin L., Kobinger G.P., Ward A.B., Saphire E.O. (2014). Structures of protective antibodies reveal sites of vulnerability on Ebola virus. Proc. Natl. Acad. Sci. USA.

[34] Tran E.E.H., Simmons J.A., Bartesaghi A., Shoemaker C.J., Nelson E., White J.M., Subramaniam S. (2014). Spatial localization of the Ebola Virus Glycoprotein Mucin-Like Domain determined by Cryo-Electron Tomography. J. Virol..

[35] Lindenbach B.D., Rice C.M. (2003). Molecular biology of flaviviruses. Adv. Virus Res..

[36] Modis Y., Ogata S., Clements D., Harrison S.C. (2004). Structure of the dengue virus envelope protein after membrane fusion. Nature.

[37] Carr C.M., Kim P.S. (1993). A spring-loaded mechanism for the conformational change of influenza hemagglutinin. Cell.

[38] Carr C.M., Chaudhry C., Kim P.S. (1997). Influenza hemagluttinin is spring-loaded by a metastable native conformation. Proc. Natl. Acad. Sci. USA.

[39] White J.M., Delos S.E., Brecher M., Schornberg K. (2008). Structures and mechanisms of viral membrane fusion proteins. Crit. Rev. Biochem. Mol. Biol..

[40] Wang H., Shi Y., Song J., Qi J., Lu G., Yan J., Gao G. (2016). Ebola viral Glycoprotein bound to its endosomal receptor Niemann-Pick {C1}. Cell.

[41] Dube D., Schornberg K.L., Shoemaker C.J., Delos S.E., Stantchev T.S., Clouse K.A., Broder C.C., White J.M. (2010). Cell adhesion-dependent membrane trafficking of a binding partner for the ebolavirus glycoprotein is a determinant of viral entry. Proc. Natl. Acad. Sci. USA.

[42] Stiasny K., Heinz F.X. (2006). Flavivirus membrane fusion. J. Gen. Virol..

[43] Stiasny K., Fritz R., Pangerl K., Heinz F.X. (2011). Molecular mechanisms of flavivirus membrane fusion. Amino Acids.

[44] Moller-Tank S., Maury W. (2015). Ebola Virus entry: A curious and complex series of events. PLOS Pathog..

[45] Misasi J., Gilman M.S.A., Kanekiyo M., Gui M., Cagigi A., Mulangu S., Corti D., Ledgerwood J.E., Lanzavecchia A., Cunningham J. (2016). Structural and molecular basis for Ebola virus neutralization by protective human antibodies. Science.

[46] Dai L., Song J., Lu X., Deng Y.Q., Musyoki A.M., Cheng H., Zhang Y., Yuan Y., Song H., Haywood J. (2016). Structures of the Zika Virus Envelope Protein and its complex with a flavivirus broadly protective antibody. Cell Host Microbe.

[47] Tung C.S., McMahon B.H. (2012). A structural model of the *E. coli* PhoB Dimer in the transcription initiation complex. BMC Struct. Biol..

[48] Meng E., Pattersen E., Souch G., Huang C., Ferrin T. (2006). Tools for integrated sequence-structure analysis with UCSF Chimera. BMC Bioinform..

[49] Hess B., Kutzner C., van der Spoel D., Lindahl E. (2008). GROMACS 4: Algorithms for highly efficient, load-balanced, and scalable molecular simulation. J. Chem. Theory Comput..

[50] Pattersen E., Goddard T., Huang C., Couch G., Greenblatt D., Meng E., Ferrin T. (2004). UCSF Chimera—A visualization system for exploratory research and analysis. J. Comput. Chem..

[51] Sikic K., Tomic S., Carugo O. (2010). Systematic comparison of crystal and NMR protein structures deposited in the Protein Data Bank. Open Biochem. J..

