# Supplementary figures and images for: Structural Transition and Antibody Binding of EBOV GP and ZIKV E Proteins from Pre-Fusion to Fusion-Initiation State
Source: Biomolecules. 2018 May 10;8(2):25. doi: 10.3390/biom8020025 (PMC6022868; doi:10.3390/biom8020025)

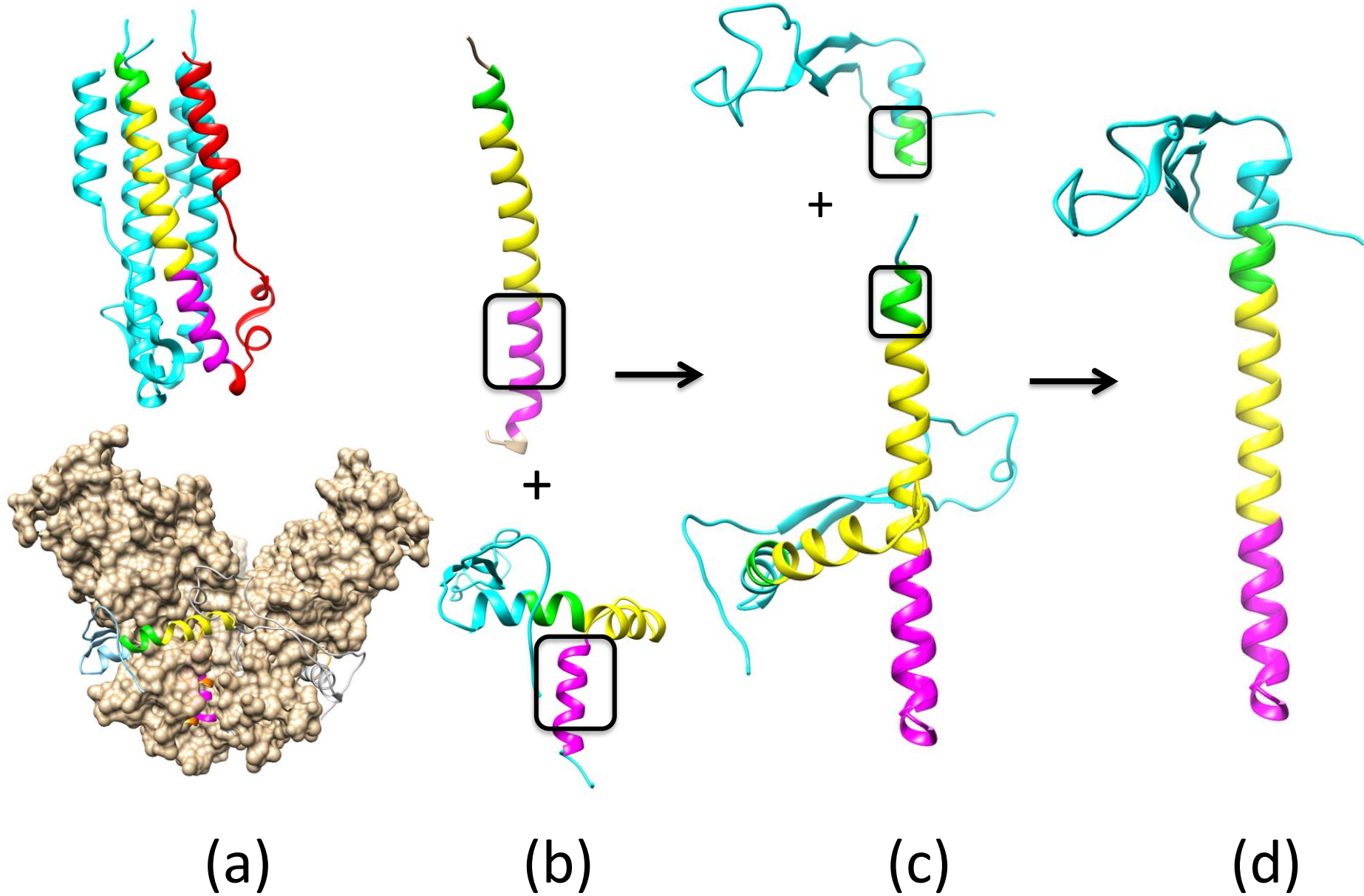

Supplement: Supplementary file 1 [file biomolecules-08-00025-s001.zip › figure-S1.pdf]

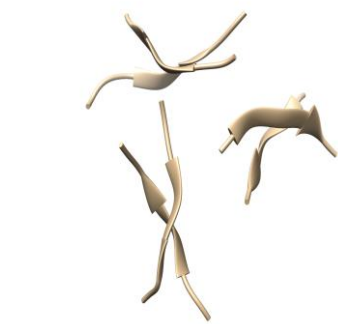

+

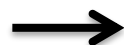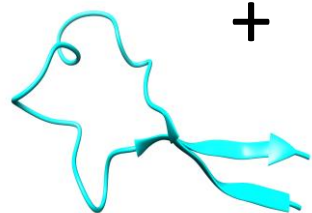

(a)

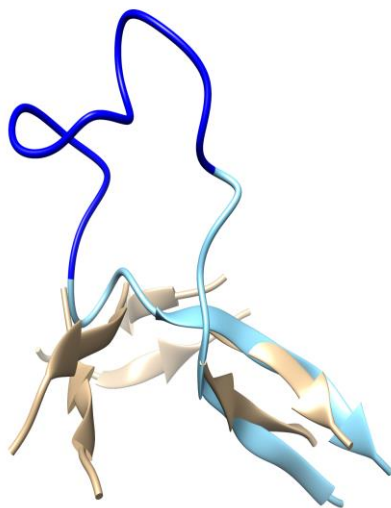

(b)

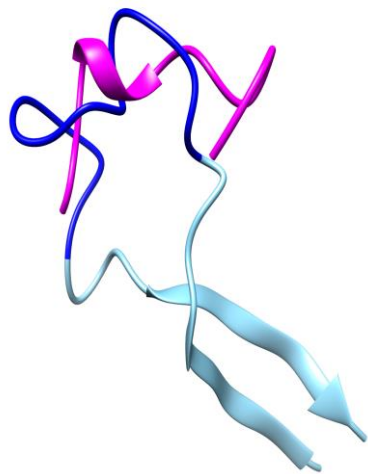

(c)

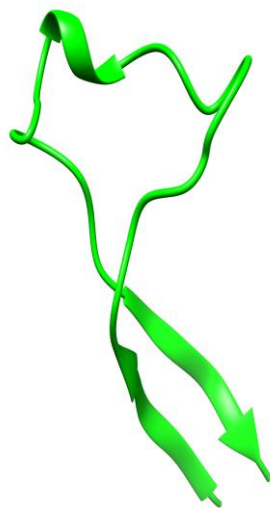

(d)

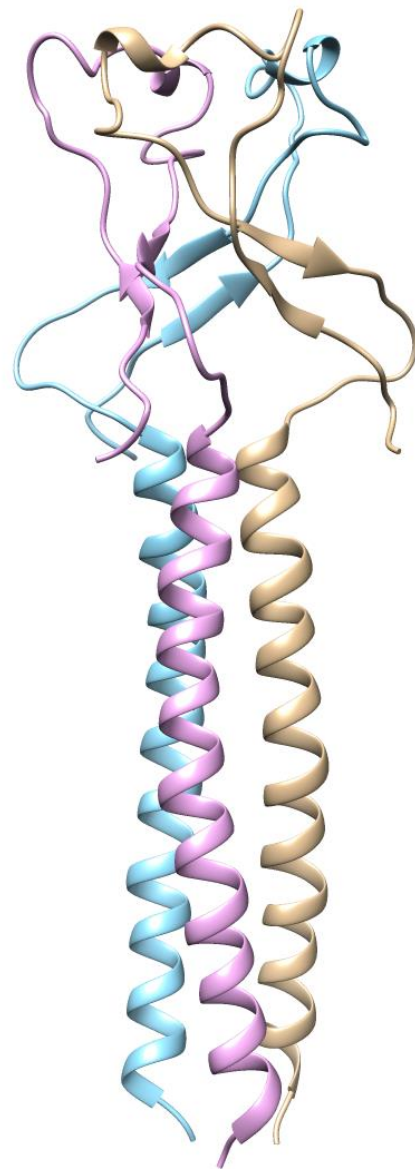

(e)

Supplement: Supplementary file 1 [file biomolecules-08-00025-s001.zip › figure-S2.pdf]

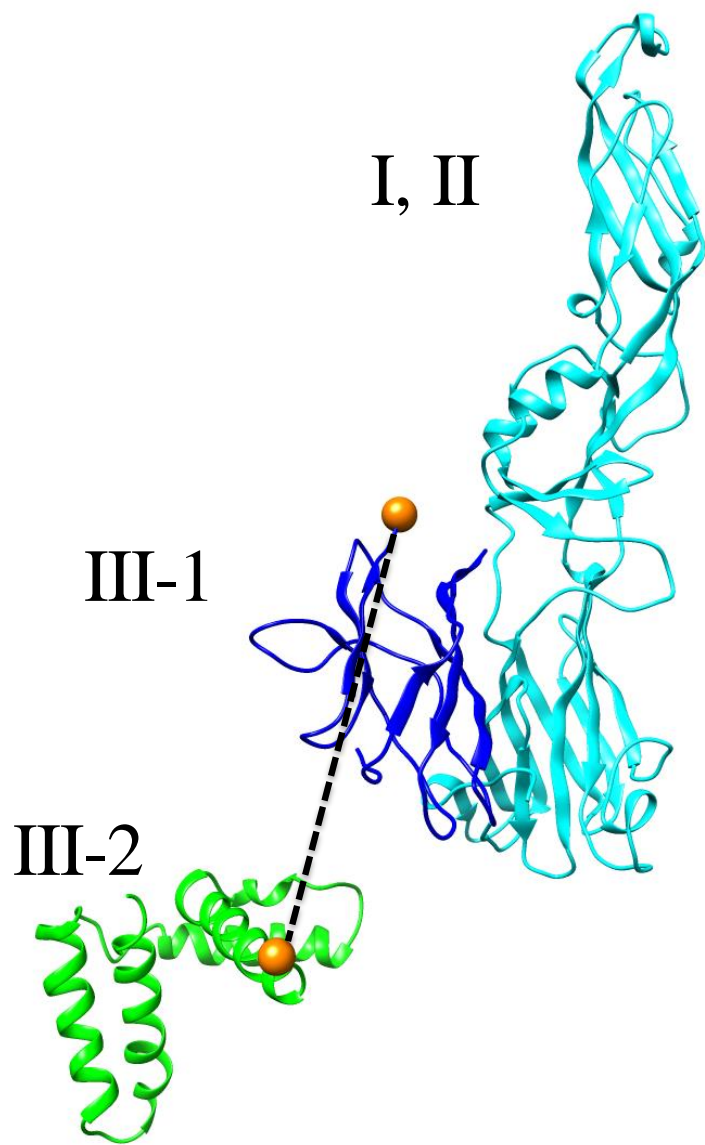

(a)

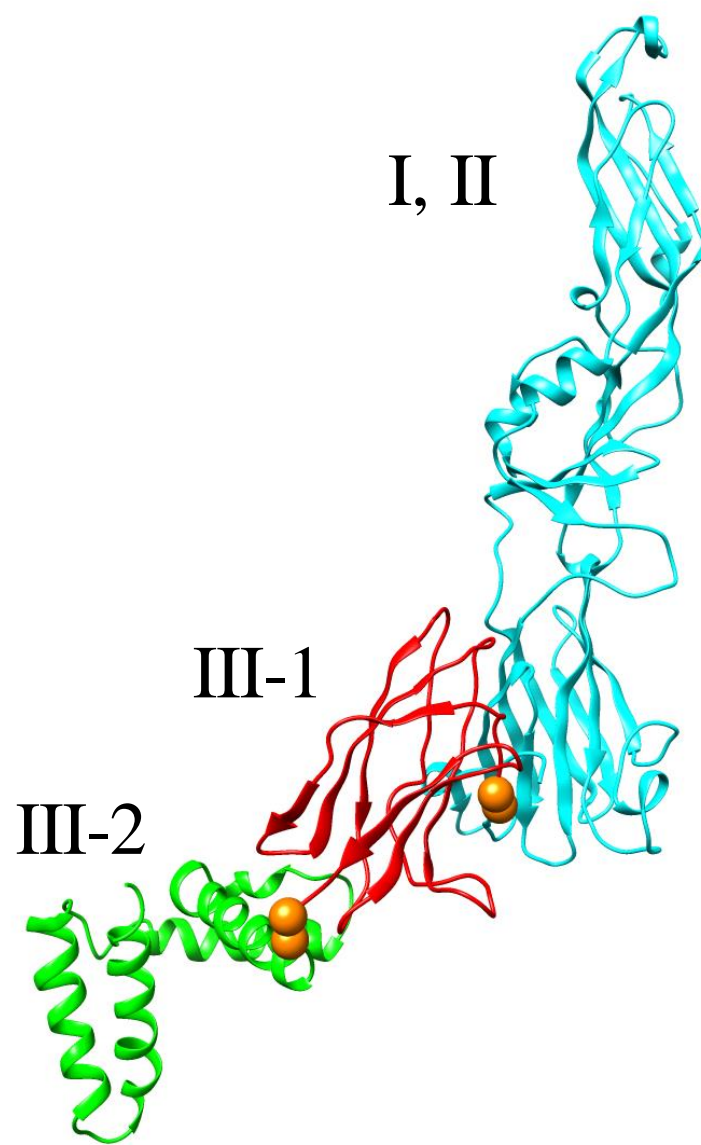

(b)

Supplement: Supplementary file 1 [file biomolecules-08-00025-s001.zip › figure-S3.pdf]

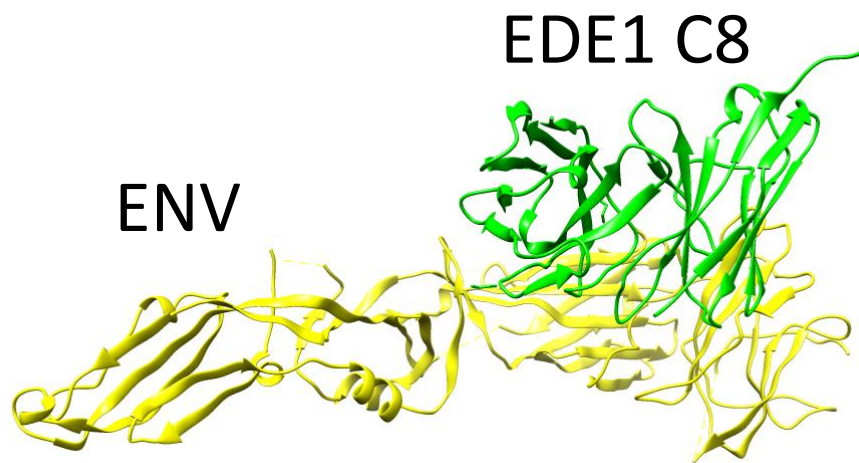

(a)

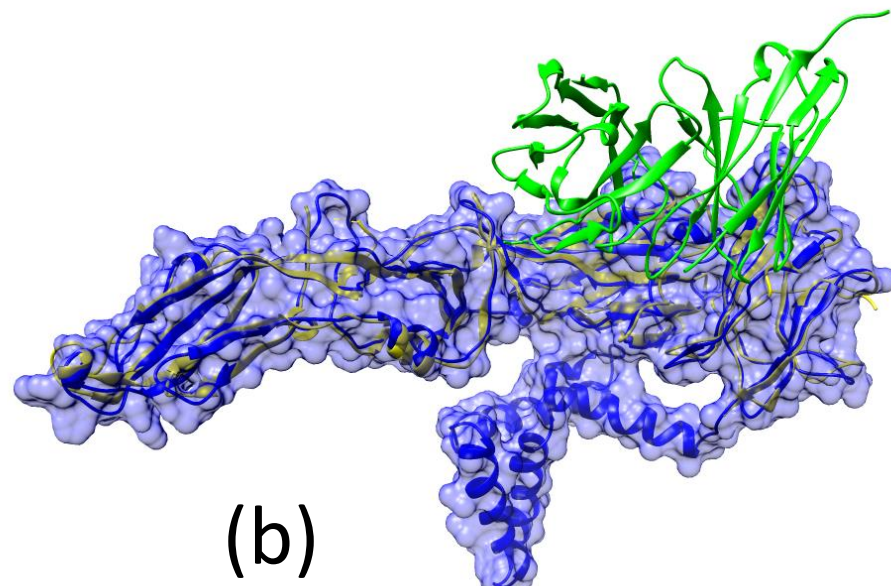

(b)

interacting  
with 2 ENVs

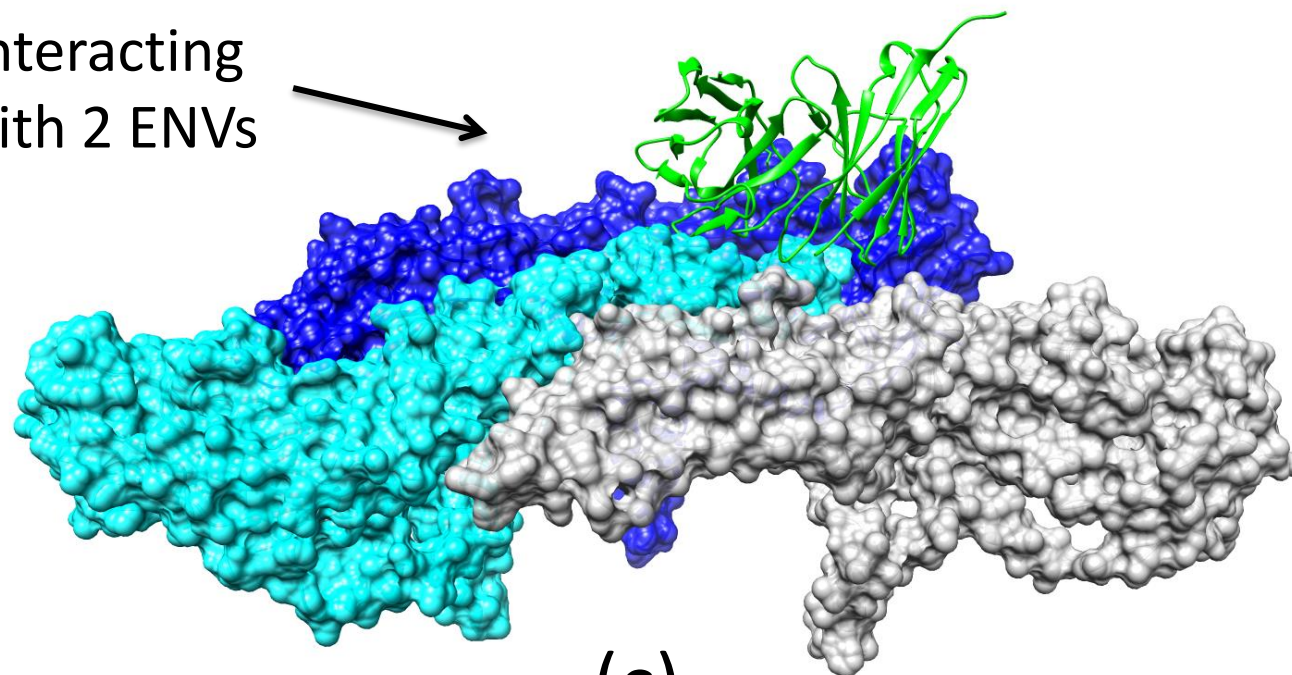

(c)

Supplement: Supplementary file 1 [file biomolecules-08-00025-s001.zip › figure-S4.pdf]
